# Deaths related to differentiated thyroid cancer: a rare but real event

**DOI:** 10.1590/2359-3997000000261

**Published:** 2017-03-20

**Authors:** Ana Kober N. Leite, Beatriz G. Cavalheiro, Marco Aurélio Kulcsar, Ana de Oliveira Hoff, Lenine G. Brandão, Claudio Roberto Cernea, Leandro L. Matos

**Affiliations:** 1 Faculdade de Medicina Universidade de São Paulo São Paulo SP Brasil Divisão de Cirurgia de Cabeça e Pescoço, Faculdade de Medicina da Universidade de São Paulo (FMUSP) e Instituto do Câncer do Estado de São Paulo (ICESP), São Paulo, SP, Brasil; 2 Faculdade de Medicina Universidade de São Paulo São Paulo SP Brasil Departamento de Endocrinologia, Faculdade de Medicina da Universidade de São Paulo (FMUSP) e Instituto do Câncer do Estado de São Paulo (ICESP), São Paulo, SP, Brasil; 3 Faculdade de Medicina Universidade de São Paulo Brasil Divisão de Cirurgia de Cabeça e Pescoço, Faculdade de Medicina da Universidade de São Paulo (FMUSP)

**Keywords:** Thyroid, thyroid cancer, metastasis, prognosis, death

## Abstract

**Objective:**

The present study describes the clinical and tumor characteristics of patients that died from differentiated thyroid cancer and reports on the cause and circumstances of death in these cases.

**Subjects and methods:**

Retrospective analysis of all the differentiated thyroid cancer (DTC) related deaths at a single institution over a 5-year period, with a total of 33 patients.

**Results:**

Most of the patients were female (63.6%), with a mean age at diagnosis of 58.2 years. The most common histologic type was papillary (66.7%) and 30.3% were follicular. The distribution according to the TNM classification was: 15.4% of T1; 7.7% T2; 38.4% T3; 19.2% of T4a and 19.2% of T4b. Forty-four percent of cases were N0; 20% N1a and 36.6% of N1b. Twelve patients were considered non-responsive to radioiodine. Only one of the patients did not have distant metastases. The most common metastatic site was the lung in 69.7%. The majority of deaths were due to pulmonary complications related to lung metastases (17 patients, 51.5%), followed by post-operative complications in 5 cases, neurological disease progression in 3 cases, local invasion and airway obstruction in one patient. Median survival between diagnosis and death was reached in 49 months while between disease progression and death it was at 22 months.

**Conclusion:**

Mortality from DTC is extremely rare but persists, and the main causes of death derive from distant metastasis, especially respiratory failure due to lung metastasis. Once disease progression is established, median survival was only 22 months.

## INTRODUCTION

Thyroid cancer (TC) is the most common endocrine malignancy and the fifth most common cancer diagnosed in women. It has been reported that its incidence has the largest annual increase in men and women amongst all cancers in the United States ( 1 ). In Brazil, it is estimated that during 2016, 1,090 new cases will be diagnosed in men and 5,870 in women, making it the 8^th^ most common cancer in women ( 2 ).

The vast majority of TCs (> 90%) originate from follicular cells and are defined as differentiated thyroid cancers (DTC) and the two histological subtypes are the papillary TC with its variants and the follicular TC ( 3 ).

Differentiated thyroid cancer is usually an indolent disease that with adequate treatment has an excellent prognosis ( 3 ). The literature reports that less than 5% of patients die from the disease within 10 years ( 4 - 6 ). However few, there are deaths related to DTC, but the rarity of this event and the long course of the disease makes it hard to analyze and determine specific risk factors for this outcome ( 7 ).

The aim of the present study was to describe the clinical and tumor characteristics of patients that died from DTC and to report the cause and circumstances of death in these cases.

## SUBJECTS AND METHODS

We conducted a retrospective cohort study at *Instituto do Câncer do Estado de São Paulo* (ICESP) amongst the 1,114 patients treated for thyroid cancer from January 2009 to November 2015. The inclusion criteria were patients with DTC that died from disease progression or complications directly related to cancer treatment. Thirty-three cases (3.0%) were identified and included in this study. The study was approved by the Institutional Review Board under the number 176/15.

From these charts data was collected and analyzed based on patient’s demographics, tumor characteristics (histologic type, size, extra-thyroid spread, recurrence, vascular invasion, node status, extra-capsular spread), treatment (surgical details, use of radioiodine, external-beam radiation, chemotherapy), outcome (recurrence, distant metastases, cause of death and the period for each outcome).

Follow-up was done according to institutional protocol: clinical examination, laboratorial analyses of thyroid hormones, thyroglobulin and anti-thyroglobulin anti-bodies every six months; neck ultrasound, chest radiography and whole body scan (selected cases) annually. Locoregional recurrence was determined when confirmed by cytological or histological analyses whereas, in distant metastasis determination, suggestive imaging studies were accepted.

The disease was considered refractory to radioiodine (RAI) therapy acording to the definition in the 2015 American Thyroid Association Guidelines: the malignant or metastatic tissue did not ever concentrate RAI, the tumor tissue lost the ability to concentrate RAI following previous evidence of RAI-avid disease, the RAI was concentrated in some lesions but not in others, and the metastatic disease progressed despite significant concentration of RAI.

For statistical analysis, SPSS^®^ version 17.0 (SPSS^®^ Inc; Illinois, USA) was used. The values obtained from the study of each continuous variable were described by means and standard-deviation (SD) and also by median and 95% confidence interval (95%CI) and relative. Absolute and relative frequencies were used to describe qualitative data. The Kaplan-Meier method was employed for survival analysis.

## RESULTS

Amongst the 33 patients that died from DTC, the majority (63.6%) were female, and the absolute majority (94.0%) of cases were diagnosed in individuals older than 45 years, with a mean age at diagnoses of 58.2 ± 12.0 years. The most common histologic type of tumor was the papillary thyroid cancer, responsible for 22 cases and 66.7% of deaths. There were 10 cases of follicular cancer and one case of not specified differentiated thyroid cancer.

Considering TNM classification, initially, most patients had T3 tumors (10 cases; 38.4%), follow by T4a and T4b with 19.2% each, four cases (15.4%) of T1 and two cases of T2 tumors. Multifocal disease was found in 50% of patients and mean tumor size was 4.7 ± 3.4 cm (minimum of 0.5 cm and maximum of 13.5 cm). Forty-four percent were N0, 20% N1a and 36% N1b. The descriptive data of the patients included in the study are described in Table 1 .

**Table 1:** Descriptive data of patients with differentiated thyroid carcinoma that died from the disease (N = 34)

Variable	N (%)
**Age**	
≥ 45 years	31 (94.0)
< 45 years	2 (6.0)
Mean ± SD	58.2 ± 12.0
**Gender**	
Male	12 (36.4)
Female	21 (63.6)
**Histologic type**	
Papillary	22 (66.7)
Follicular	10 (30.3)
**T stage**	
T1	4 (15.4)
T2	2 (7.7)
T3	10 (38.4)
T4a	5 (19.2)
T4b	
**N stage**	
N0	11 (44.0)
N1a	5 (20)
N1b	9 (36.0)
**Multifocality**	
Yes	9 (50.0)
No	9 (50.0)

N: number of cases.

Thirty-one patients were submitted to surgical treatment initially; only two cases were not because were considered inoperable from the moment of diagnosis. Thirteen patients were submitted to total thyroidectomy; eight had total thyroidectomy and central node dissection; eight had total thyroidectomy, central and lateral node dissection and two partial thyroidectomies as the initial treatment.

Twenty-two patients (66.7%) received radioactive iodine (RAI) therapy, with an average of 499.4 mCi ± 264.8 in total dose (minimum of 50mCi and maximum of 1200 mCi). Twelve cases were considered unresponsive to RAI throughout the treatment. The mean stimulated thyroglobulin was 19,755 ± 5,850 ng/mL and just one case had negative thyroglobulin during the follow-up.

Sixteen patients developed nodal recurrence and fourteen of these were submitted to a new surgical procedure. Thirty-two patients presented distant metastasis. Of these, in 42.4% the metastasis was diagnosed at the same time as the primary tumor. The most common site of metastasis was the lung (69.7%), followed by bone in 66.7%. Two patients had liver metastasis and two had kidney metastasis, but all of these also had lung spread as well. Forty-two percent of cases had metastasis in more than one site. During disease progression nine cases were considered to have dedifferentiation from the initial tumor.

Only eight patients did not receive treatment specifically for the metastasis or its complications. Ten were submitted to surgery, six received sorafenib as part of a clinical trial, eleven underwent radiation therapy and six chemotherapy.

When analyzing the specific cause that lead to their deaths, it was established that the majority of patients died from respiratory failure related to their lung metastasis (17 patients, 51.5%). Other causes were post-operative complications in 5 cases, neurological disease progression in 3 cases (central nervous system invasion), local invasion and airway obstruction in one patient and other causes in 7 patients. The absolute majority of patients (85.3%) died either from progression of their metastasis or complications derived directly from its treatment.

**Figure 1:**
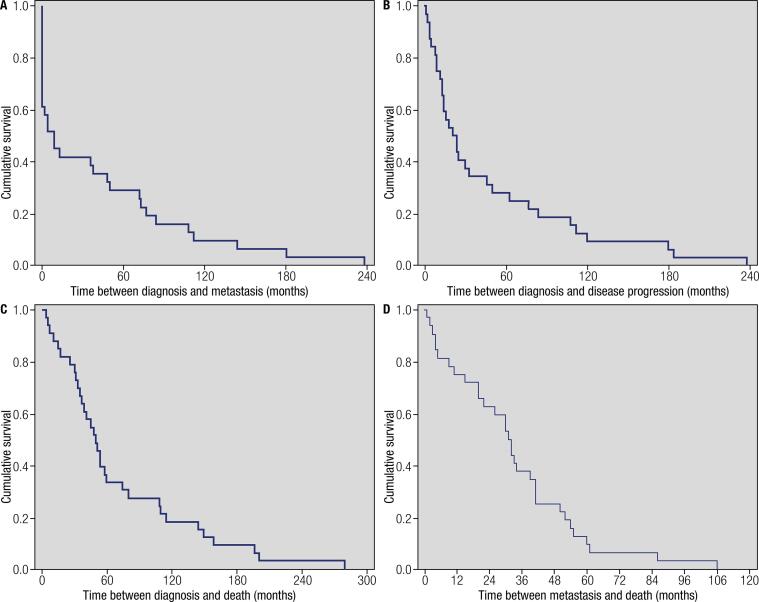
Kaplan-Meier curves demonstrating the evolution of cumulative survival between diagnosis and metastatic disease (A), between the diagnosis and disease progression (B), between diagnosis and death (C) and between metastasis diagnosis and death (D) in the evolution of the disease in patients that died from differentiated thyroid carcinoma.

**Figure 2:**
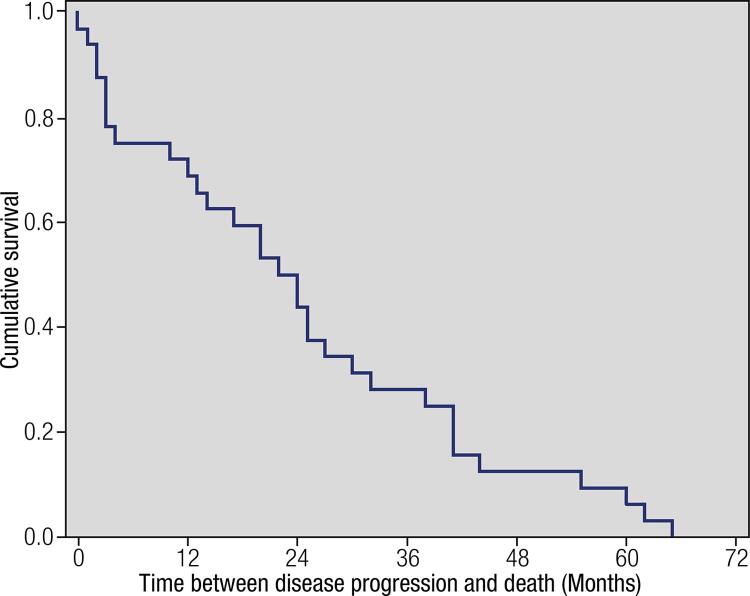
Kaplan-Meier curves demonstrating a short time between disease progression and death in patients with differentiated thyroid carcinoma (median survival reached in 22 months).

The mean time between diagnosis and death was of 71.4 months (median 49 months, minimum of 4 months and maximum of 279 months). The survival analysis ( Figure 1 ) revealed that the great majority of patients have low cumulative survival in the first 5 years of follow-up. Median survival between diagnosis and first evidence of metastatic disease was reached in 9 months (CI95%: 0.0 – 20.9 months), between the diagnosis and disease progression in 21 months (CI95%: 9.9 – 32.1 months), between diagnosis and death in 49 months (CI95%: 36.8 – 61.2 months) and between first metastasis diagnosis and death in 31 months (CI95%: 24.3 – 37.6 months). Moreover, the median survival was reached in 22 months (CI95%: 14.2 – 29.8 months) from the disease progression until death ( Figure 2 ).

## DISCUSSION

The present study identified a death rate of 3.0% in a cohort of 1,114 patients treated for differentiated thyroid cancer in a single institution, one of the largest casuistic in the literature. Moreover, the majority of these patients had advanced disease, almost all of them with distant metastasis, especially pulmonary. Some developed RAI therapy refractory disease, tumor dedifferentiation and most patients quickly developed disease progression, despite the therapeutic approach. We also found that once disease progression has been established, the mean survival drops significantly and within 22 months of disease progression 50% of the patients are dead.

Physicians consider differentiated thyroid cancer an indolent disease that will have a good outcome if correctly treated at diagnoses. As a result, there is a global tendency to treat DTC less aggressively over the years ( 8 - 12 ). However, since its extremely low mortality is balanced by its high prevalence, the number of deaths cannot be overlooked. The American Cancer Society estimates over 2,800 deaths related to DTC in 2014 ( 13 ).

Similarly to the present study that demonstrated a death rate of 3.0%, mortality related to DTC is reported to be lower than 5% at 10 years ( 4 - 6 , 14 ). Because it is a rare outcome in a disease with long clinical course it is extremely difficult to analyze the factors that may lead to it prospectively, which makes reports like the present one become more significant.

Initial reports on this subject from the 1960s show that local disease progression with airway obstruction was a significant cause of death, almost as much as distant metastasis. Tollefsen and cols. ( 15 ) in 1964 reported 40% of deaths from thyroid cancer related to local disease progression and 52% related to distant metastasis. Smith and cols. ( 16 ) at Mayo Clinic reported a similar scenario in 1988, with 36% of deaths from local disease progression and 37% from lung metastasis progression.

However, this distribution changed in the series closer to 21^st^ Century and deaths due to local disease progression became rare. In 2001, Beasley and cols. ( 17 ) showed 70% of deaths related to distant metastasis and 20% from local recurrence. Nixon and cols. ( 7 ) reported on 17 deaths from DTC with 88% of deaths from distant metastasis and two from aspiration pneumonia that could be related to local disease complications. These findings are similar to the results reported in the present study, that show 85.3% of deaths related to distant metastasis progression and only one case (3%) from local disease progression.

This shift on the specific cause that leads to death in DTC patients can most certainly be explained by the change in treatment approach in the last decades, with more aggressive surgical therapy to remove all gross disease, including airway and esophagus resection if necessary. Also, radioiodine treatment is more available and used in the majority of cases, which probably contributes to this shift in disease progression.

Studies associate deaths from DTC with extra-thyroid extension, nodal disease, age over 45 years and distant metastasis ( 7 , 16 , 17 ). In our series the majority of patients had age over 45 years, nodal disease and all but one patient had distant metastasis.

Many cases had advanced disease at presentation, with 42.5% of metastasis already at initial diagnosis and 60% of T3 and T4 tumors. The literature also shows that advanced disease is usually present at the initial diagnosis in the patients that will die from DTC ( 7 , 17 ).

**Figure 3:**
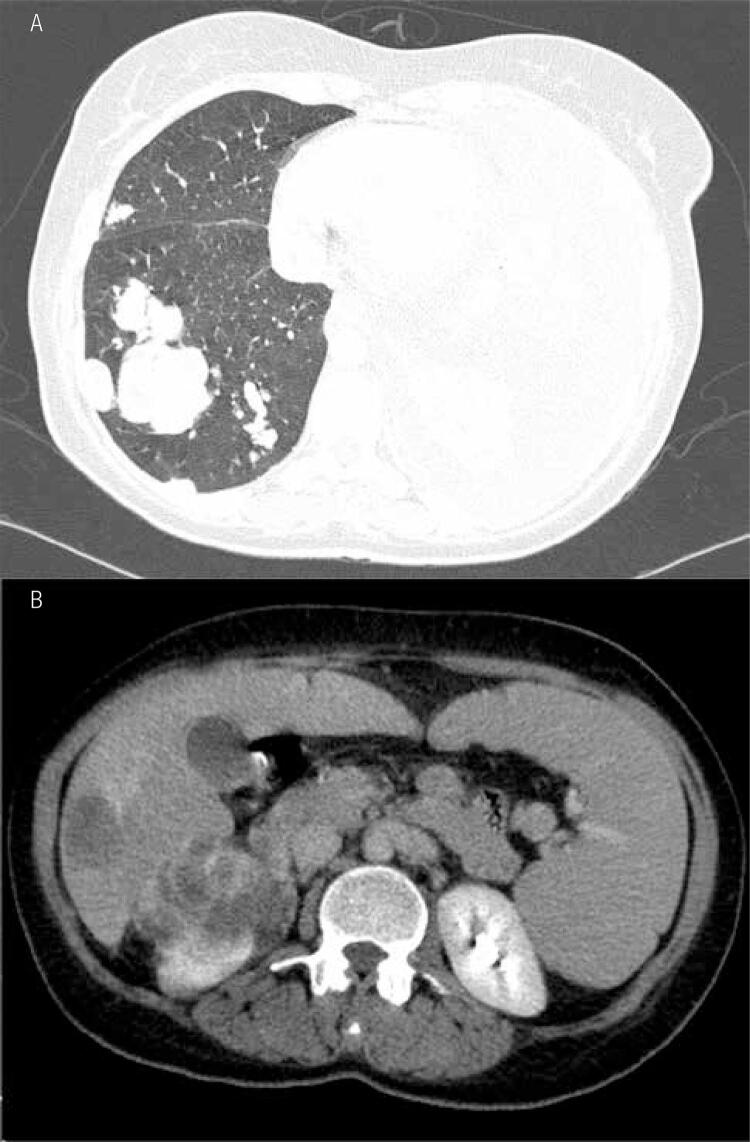
Image of chest CT from a female patient with a pT1a follicular carcinoma of 5 mm with pulmonary disease that died from obstruction of the left bronchus (A) by a metastasis six years after initial diagnosis. The patient presented also liver and kidney metastasis (B).

However, there are some cases that do not follow this pattern. In this series two patients presented with initial tumors, no nodal or distant metastasis, and during follow-up developed lung metastasis that eventually led to their deaths. One was a 63 year-old male with a 1.3 cm papillary TC that had a nodal recurrence after two years of thyroidectomy, as well as lung metastasis four years after initial diagnosis, and died within one year. The other was a 42 year-old female with a 0.5 cm unifocal folicullar carcinoma and no nodal metastasis that developed lung metastasis after four years of surgery and died six years after initial diagnosis from lung disease progression ( Figure 3 ).

Nilubol and Kebebew ( 13 ) identified, using the database of SEER (Surveillance, Epidemiology, and End Results), that amongst 1,753 deaths from DTC 12.3% were tumors with less than 2.0 cm, which brings directly into question the safety of non-operative management of initial DTC that has recently been advocated by some authors ( 11 ). Our findings of two cases with less than 2.0 cm in 33 deaths from DTC corroborate that the non-operative approach should be treated with caution.

In conclusion, mortality from DTC is extremely rare but persists, and the main cause of death is respiratory failure due to lung metastasis. The absolute majority of patients die from distant metastasis with rapid progression and present with advanced disease, but there are cases of initial indolent disease that later develop metastasis that lead to death. The very low mortality by DTC makes this a hard subject to study. There are only small series available in the literature with distinct inclusion criteria and methodologies and, therefore, it is not possible to make any useful conclusions as to what makes some DTC behave so aggressively. Further studies in molecular biology are necessary to establish factors associated with aggressiveness in DTC so that we can select the patients at high risk for mortality and treat them accordingly.

This study has the limitation of being a retrospective descriptive analyses of a cohort of patients that died from DTC, therefore preventing further analyses of factors related to this outcome. Prospective trials would bring very useful knowledge on this subject, but are very hard to do since mortality is very low and disease clinical course is long. Also, because it was not a prospective study, follow up and scans intervals may have varied between patients.
